# Handling Multi-Source Uncertainty in Accelerated Degradation Through a Wiener-Based Robust Modeling Scheme

**DOI:** 10.3390/s25216654

**Published:** 2025-10-31

**Authors:** Hua Tu, Xiuli Wang, Yang Li

**Affiliations:** 1 School of Mechatronic Engineering and Automation, Shanghai University, Shanghai 200444, China; 2 College of Information Engineering, Zhejiang University of Technology, Hangzhou 310023, China; 3 College of Software, Nankai University, Tianjin 300350, China

**Keywords:** heterogeneous degradation paths, accelerated lifetime modeling, robust framework, interval-based inference

## Abstract

Uncertainty from heterogeneous degradation paths, limited experimental samples, and exogenous perturbations often complicates accelerated lifetime modeling and prediction. To confront these intertwined challenges, a Wiener process-based robust framework is developed. The proposed approach incorporates random-effect structures to capture unit-to-unit variability, adopts interval-based inference to reflect sampling limitations, and employs a hybrid estimator, combining Huber-type loss with a Metropolis–Hastings algorithm, to suppress the influence of external disturbances. In addition, a quantitative stress–parameter linkage is established under the accelerated factor principle, supporting consistent transfer from accelerated testing to normal conditions. Validation on contact stress relaxation data of connectors confirms that this methodology achieves more stable parameter inference and improves the reliability of lifetime predictions.

## 1. Introduction

Reliability assessment under accelerated degradation testing (ADT) is often challenged by multiple forms of uncertainty, including internal heterogeneity, limited test data, and external perturbations [1,2]. To obtain credible inference under these conditions, modeling strategies capable of addressing such variability are required. In the ADT literature, three principal methodological paradigms can be identified: physics-based models, data-driven algorithms, and stochastic process-based approaches [3,4,5]. Physics-oriented models attempt to embed material behavior and stress–strain mechanisms into the formulation, yet their applicability is limited when detailed mechanistic information is incomplete or unavailable [6]. Data-centric approaches, including recent advances in machine learning and statistical regression, provide flexibility but generally suffer from reduced interpretability and weak extrapolation for long-term reliability prediction [7,8].

In recent years, advanced data-driven techniques have achieved remarkable progress in condition monitoring and remaining useful life (RUL) prediction. Representative examples include attention-guided graph isomorphism networks for capturing spatial–temporal dependencies in equipment degradation signals [9], progressive contrastive representation learning frameworks for fault diagnosis under limited labels [10], and comparative studies on blind diagnostic indicators for intelligent condition monitoring [11]. These approaches have demonstrated strong feature-extraction capability and robustness to heterogeneous data sources. In [12], an adaptive spatiotemporal graph attention network with Decision-Making Trial and Evaluation Laboratory (DEMATEL)-based relation mining was proposed for accurate RUL prediction of aviation equipment. A self-supervised progressive learning (SSPL) framework was proposed in [13] to enhance machinery fault diagnosis by dynamically generating pretext tasks from easy to difficult, achieving high accuracy under limited labeled data and varying conditions. Jachymczyk et al. [14] proposed a structured feature selection approach for predictive maintenance of wind turbine blades, which integrates correlation analysis and visual evaluation to eliminate redundant condition indicators and enhance classification efficiency while maintaining diagnostic accuracy. However, most of them emphasize feature-level health assessment rather than explicit lifetime modeling, and their extrapolation from observed degradation data to reliability metrics under accelerated conditions remains challenging.

By contrast, stochastic process models strike a balance between theoretical rigor and practical adaptability, offering a mathematically tractable means to characterize randomness in degradation trajectories [15,16]. This capability makes stochastic process-based methods an attractive option for robust lifetime modeling and reliability evaluation in ADT studies. Within the stochastic process framework, the Wiener process [17], Gamma process [18], and inverse Gaussian process [19] are the most widely adopted tools for modeling monotonic or diffusive degradation. For instance, Si et al. ([17]) proposed a Wiener process-inspired semi-stochastic filtering approach that estimates parameters via maximum likelihood and enables probabilistic remaining useful life (RUL) prediction by integrating historical and real-time data. Wang et al. ([20]) proposed inference procedures for the Gamma degradation model with random effects, while Zhou et al. ([21]) developed optimal design procedures for constant-stress ADT under inverse Gaussian process models, emphasizing measurement timing as a key variable and deriving analytical and numerical solutions that improve test efficiency through optimal time scheduling. More recently, Lu et al. [5] investigated a mixed process-based reliability analysis using a series of stochastic process models, demonstrating its flexibility in handling misspecification and structural differences. Other processes, such as Lévy processes, have also been explored to capture heavy-tailed jumps and non-Gaussian behaviors, yet their application remains limited due to mathematical intractability and the lack of closed-form analytical expressions [22]. Comparatively, the Gamma and inverse Gaussian processes are effective for strictly monotonic degradation, but they may not fully capture measurement fluctuations. By contrast, the Wiener process, with its additive Gaussian noise structure, is well-suited for modeling degradation subject to small perturbations and provides a convenient likelihood formulation. Motivated by these advantages, this paper adopts the Wiener process as the core stochastic model.

Nevertheless, several challenges remain in accelerated degradation modeling. In practice, degradation trajectories are simultaneously affected by three categories of uncertainty [18,23]. The first is internal variability caused by heterogeneity, which originates from design deviations, manufacturing tolerances, and process imperfections, thereby producing dispersion in product performance [24,25]. The second is uncertainty associated with limited sample sizes, where the scarcity of experimental observations reduces estimation stability and inflates parameter variability [26]. The third is external disturbance, including measurement noise, sensor failures, or even intentional attacks such as cyber intrusions, all of which distort the collected degradation signals [27,28,29]. Overlooking any of these sources of uncertainty can lead to biased inference and unreliable lifetime assessment. Furthermore, the classical assumption proposed by Pieruschka, that variations in stress levels merely translate into parameter changes, raises a fundamental challenge: identifying which parameters are stress-dependent and clarifying how they vary with stress [30]. Quantitatively resolving this stress–parameter linkage remains crucial for credible extrapolation from accelerated to nominal stress conditions.

Based on the above description, the limitations can be summarized as follows:

- Incomplete handling of hybrid uncertainties, including internal heterogeneity, limited sample size, and external disturbances.
- Lack of robustness in parameter estimation when degradation data contain multi-source uncertainties.
- Insufficient quantitative linkage between stress and model parameters, leading to biased extrapolation from accelerated to nominal conditions.
- Limited integration of robust statistical inference and stochastic degradation modeling within a unified reliability analysis framework.

To overcome the above challenges, this study develops a Wiener process-based accelerated degradation modeling framework with enhanced robustness. Internal variability is addressed by embedding random effects into the model structure, which captures unit-to-unit heterogeneity. The instability caused by small sample sizes is alleviated through interval estimation, allowing parameter inference to explicitly reflect sampling-induced uncertainty. External perturbations are controlled by a hybrid estimation strategy that integrates Huber-type loss with a Metropolis–Hastings sampler, thereby reducing sensitivity to outliers and non-Gaussian noise. In addition, the accelerated factor constant principle is employed to construct an explicit stress–parameter linkage, enabling consistent transfer of model estimates from accelerated conditions to nominal operating environments. Application to connector stress relaxation data demonstrates that the proposed approach yields more credible parameter estimation and improves reliability predictions under practical scenarios.

The main contributions of this study are summarized as follows:

- A robust stochastic degradation modeling framework is established on the basis of a Wiener-process formulation, where random effects are incorporated to capture heterogeneity. In addition, interval estimation is introduced to explicitly quantify the uncertainty induced by limited sample sizes, thus improving the credibility of parameter inference under small-sample conditions.
- A hybrid estimation approach is proposed that integrates Huber-type loss with a Metropolis–Hastings sampler, enhancing robustness against measurement noise, outliers, and other external disturbances.
- An explicit stress–parameter relationship is formulated under the accelerated factor constant principle, providing a systematic means to extrapolate accelerated test results to nominal operating conditions for reliability and lifetime analysis.

The remainder of this paper is organized as follows. Section 2 introduces the Wiener process-based accelerated degradation model, in which random effects are employed to capture internal uncertainty caused by heterogeneity. Section 3 develops the stress–parameter relationship under the accelerated factor constant principle and derives the corresponding lifetime distribution. Section 4 details the interval estimation and the hybrid inference procedure that integrates Huber-type loss with a Metropolis–Hastings algorithm to mitigate the influence of external disturbances. Section 5 illustrates the effectiveness of the proposed approach using stress relaxation data from electrical connectors. Finally, Section 6 concludes the study and highlights the methodological contributions.

## 2. The Accelerated Degradation Model Under Internal Uncertainty

**Figure 1:**
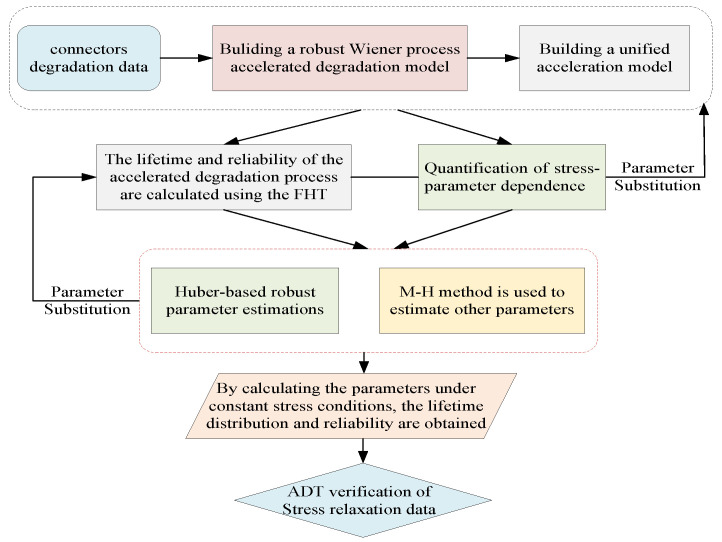
The flowchart of the methodological framework.

Internal uncertainty is intrinsic to the degradation behavior of electrical connectors, primarily manifesting as population heterogeneity caused by design tolerances and manufacturing variability. This paper focuses on the multi-source uncertainties inherent in such products and addresses these issues by constructing a robust Wiener process modeling framework. The overall flowchart of the proposed approach is shown in Figure 1.

This methodological framework explicitly outlines the four key stages: (i) ADT model formulation with random effects, (ii) stress–parameter linkage derivation under the accelerated factor principle, (iii) robust parameter estimation integrating Huber loss and the Metropolis–Hastings algorithm, and (iv) lifetime and reliability analysis. To rigorously account for such variability, this section develops a Wiener process-based degradation model augmented with random effects to characterize unit-to-unit differences in reliability behavior.

### 2.1. The Wiener Process-Based ADT Model

The Wiener process offers a flexible and analytically tractable stochastic framework that has been widely adopted in reliability modeling [31]. Unlike strictly monotonic processes, it accommodates both deterministic drift and stochastic fluctuations, making it particularly suitable for degradation phenomena influenced by random perturbations. This dual capability allows the Wiener process to capture gradual deterioration while realistically reflecting measurement noise and operational variability, which are inevitable in complex systems.

When internal heterogeneity is introduced into the degradation dynamics, the Wiener process becomes especially advantageous. Through its drift component, it represents the systematic degradation trend, while the diffusion term naturally incorporates stochastic variability across units. This property makes it well-suited for systems in which degradation is not strictly monotonic but may fluctuate due to intrinsic randomness or data acquisition disturbances. By explicitly modeling both trend and uncertainty, the Wiener process provides a robust basis for predicting a component’s remaining useful life under varying stress conditions.

For a single component subject to ADT, the time-dependent degradation trajectory X(t) can be expressed as a Wiener process with drift,

(1) $$X\left(t\right)=W\left(s\right)t+\sigma_{w}B\left(t\right)$$

where W(s) is the drift parameter governing the average degradation rate, $\sigma_{w}$ is the diffusion coefficient that controls the magnitude of random fluctuations, and B(t) denotes a standard Brownian motion such that B(t)∼N(0,t),∀t≥0. This formulation enables flexible modeling of degradation under accelerated stress and offers a rigorous foundation for extrapolating lifetime predictions to normal operational environments. To account for internal uncertainty, we introduce a random effect into Equation (1). Specifically, let the drift parameter W(s) follow a normal distribution, $W\left(s\right)∼N\left(\mu ,\sigma^{2}\right)$, where μ denotes the time–stress-dependent structure and $\sigma^{2}$ captures unit-to-unit variations.

According to the above properties, the process X(t) in Equation (1) has the independent increment *x*, and for each time increment ΔΛ(t), the corresponding increment can be expressed as ([24]):

(2) $$\begin{array}{cc} x & =X(\Lambda (t)+\Delta \Lambda (t))-X(\Lambda (t))\\ \; & =W\left(s\right)\left(\Lambda \left(t\right)+\Delta \Lambda \left(t\right)\right)+\sigma_{w}B\left(\Lambda \left(t\right)+\Delta \Lambda \left(t\right)\right)-\left[W\left(s\right)\Lambda \left(t\right)+\sigma_{w}B\left(\Lambda \left(t\right)\right)\right]\\ \; & =W\left(s\right)\Delta \Lambda \left(t\right)+\sigma_{w}\left[B(\Lambda (t)+\Delta \Lambda (t))-B(\Lambda (t))\right] \end{array}$$

where Λ(t) is a nonnegative function, which can be specified as $\Lambda \left(t\right)=t^{c}$ with c>0. By combining the facts that $W\left(s\right)∼N\left(\mu ,\sigma^{2}\right)$ and B(t)∼N(0,Λ(t)), one obtains that $x∼N\;\left(\mu \Delta \Lambda \left(t\right),\;\sigma^{2}\Delta \Lambda {\left(t\right)}^{2}+\sigma_{w}^{2}\Delta \Lambda \left(t\right)\right)$. Therefore, the probability density function (PDF) of *x* takes the form:

(3) $$f\left(x\right)=\frac{1}{\sqrt{2\pi \Delta t\left(\sigma^{2}\Delta t+\sigma_{w}^{2}\right)}}\exp\left[-\frac{{\left(x-\mu \Delta t\right)}^{2}}{2\Delta t(\sigma^{2}\Delta t+\sigma_{w}^{2})}\right]$$

where, without ambiguity, the time increment is represented as Δt=ΔΛ(t). Therefore, the unknown parameter vector associated with Equation (3) is defined as $\theta_{w}=\left\{\mu ,\sigma ,\sigma_{w},c\right\}$. For the ADT model, it is also necessary to establish a parameter–stress dependence mapping.

### 2.2. Formulations of Acceleration Models

ADT modeling typically consists of two main parts. The above section described the degradation process, while the present section focuses on the acceleration mechanism. The role of an acceleration model is to describe how stress intensity *S* influences the degradation rate *R*. Three typical functional forms are extensively employed:

(4) $$R_{A}=\lambda_{0}\exp\;\left(-\frac{\lambda_{1}}{S}\right)$$

(5) $$R_{P}=\lambda_{0}S^{\lambda_{1}}$$

(6) $$R_{E}=\lambda_{0}\exp\left(\lambda_{1}S\right)$$

To facilitate comparison, these expressions can be recast into a unified linear form after applying a logarithm:

(7) $$\ln R=h_{0}+h_{1}\varphi \left(S\right)$$

Depending on the specific acceleration law, the terms are specified as follows:

- Arrhenius: $h_{0}=\ln\lambda_{0},\;h_{1}=-\lambda_{1},\;\varphi \left(S\right)=1/S$,
- Power law: $h_{0}=\ln\lambda_{0},\;h_{1}=\lambda_{1},\;\varphi \left(S\right)=\ln S$,
- Exponential: $h_{0}=\ln\lambda_{0},\;h_{1}=\lambda_{1},\;\varphi \left(S\right)=S$.

For practical use, φ(S) is often normalized so that its value ranges between 0 and 1:

(8) $$\varphi \left(S\right)=\left\{\begin{array}{cc} \frac{1/S_{0}-1/S}{1/S_{0}-1/S_{H}}, & \mathrm{Arrhenius}\;\mathrm{case},\\ \frac{\ln S-\ln S_{0}}{\ln S_{H}-\ln S_{0}}, & \mathrm{Power}\;\mathrm{law}\;\mathrm{case},\\ \frac{S-S_{0}}{S_{H}-S_{0}}, & \mathrm{Exponential}\;\mathrm{case}. \end{array}\right.$$

where $S_{0}$, *S*, and $S_{H}$ denote the baseline, the applied, and the highest stress levels, respectively. The transformation guarantees $\varphi \left(S_{0}\right)=0,\varphi \left(S_{H}\right)=1$. Some details can be found in [5].

According to the Pieruschka hypothesis, most existing parameter–stress dependence mapping approaches rely on assumptions derived from engineering practice or expert judgment. Based on these assumptions, the following cases can be considered:

1. **Single-parameter assumption**: Each of the unknown parameters $\mu ,\sigma ,\sigma_{w}$ is assumed to depend on stress individually, while the other two remain constant.
2. **Two-parameter assumption**: Any two of the parameters $\mu ,\sigma ,\sigma_{w}$ are assumed to be stress-dependent, with the remaining one independent of stress.
3. **Three-parameter assumption**: All three parameters $\mu ,\sigma ,\sigma_{w}$ are assumed to vary with stress.

It should be emphasized that the time-shift parameter *c* is not included in these scenarios and is treated as stress-independent. Therefore, based on these assumptions, we can obtain the following parameter–stress relationships:

1. **Single-parameter assumption**
  (a) $\ln\mu =a_{\mu }+b_{\mu }\varphi \left(S\right)$, while σ and $\sigma_{w}$ remain constant.
  (b) $\ln\sigma =c_{\sigma }+d_{\sigma }\varphi \left(S\right)$, while μ and $\sigma_{w}$ remain constant.
  (c) $\ln\sigma_{w}=u_{w}+v_{w}\varphi \left(S\right)$, while μ and σ remain constant.
2. **Two-parameter assumption**
  (a) $\ln\mu =a_{\mu }+b_{\mu }\varphi \left(S\right),\;\ln\sigma =c_{\sigma }+d_{\sigma }\varphi \left(S\right)$, while $\sigma_{w}$ remains constant.
  (b) $\ln\mu =a_{\mu }+b_{\mu }\varphi \left(S\right),\;\ln\sigma_{w}=u_{w}+v_{w}\varphi \left(S\right)$, while σ remains constant.
  (c) $\ln\sigma =c_{\sigma }+d_{\sigma }\varphi \left(S\right),\;\ln\sigma_{w}=u_{w}+v_{w}\varphi \left(S\right)$, while μ remains constant.
3. **Three-parameter assumption**
  $\ln\mu =a_{\mu }+b_{\mu }\varphi \left(S\right),\;$$\ln\sigma =c_{\sigma }+d_{\sigma }\varphi \left(S\right),\;$$\ln\sigma_{w}=u_{w}+v_{w}\varphi \left(S\right)$.

## 3. Lifetime Analysis and Characterization of Stress–Parameter Relations

After defining the stochastic degradation model, the subsequent task is to characterize the lifetime distribution through the first hitting time (FHT) and to examine how the parameters depend on stress conditions.

### 3.1. Formulation of Lifetime Distribution

In stochastic degradation modeling, the notion of FHT is central: it describes the earliest time at which a degradation trajectory crosses a critical failure boundary. This probabilistic description directly reflects the random variability of component lifetimes and provides a theoretical basis for remaining useful life estimation and maintenance scheduling [32].

Let *T* denote the random lifetime, defined by the condition that the latent degradation path Y(t) first reaches the threshold ω:

(9) T=inf{t≥0:Y(t)≥ω}

For Wiener-type processes, the distribution of *T* can be related to the hitting probability of the degradation path. In particular, the CDF takes the form

(10) $$F_{T}\left(t\right)=P\left(T\leq t\right)=P\left(Y\left(t\right)\geq \omega \right)=1-F_{W}\left(\omega ;\;\mu ,\;\sigma ,\sigma_{w}\right)$$

where $F_{W}\left(\cdot \right)$ denotes the distribution function of the corresponding Wiener law.

More explicitly, the FHT distribution of a Wiener process with positive drift μ admits the closed-form expression [24]

(11) $$F_{T}\left(t\right)=1-\Phi \left(\frac{\omega -\mu t}{\sqrt{\sigma_{w}^{2}t+\sigma^{2}t^{2}}}\right)+\exp\left(\frac{2\mu \omega }{\sigma_{w}^{2}}+\frac{2\sigma^{2}\omega^{2}}{\sigma_{w}^{4}}\right)\Phi \left(-\frac{2\sigma^{2}\omega t+\sigma_{w}^{2}\left(\mu t+\omega \right)}{\sigma_{w}^{2}\sqrt{\sigma_{w}^{2}t+\sigma^{2}t^{2}}}\right)$$

with Φ(·) denoting the standard normal cumulative distribution function. The additional term on the right-hand side captures the fact that, in a Wiener process, the event $\left\{X\left(t\right)\leq \omega \;\mathrm{and}\;X^{*}\left(t\right)>\omega \right\}$ occurs with positive probability [23], where $X^{*}\left(t\right)=\sup_{s\in [0,t]}X\left(s\right)$. As a result, the conditions T>t and X(t)≤ω cannot be regarded as equivalent. Nevertheless, whenever $\mu t\geq \sqrt{\sigma_{w}^{2}t+\sigma^{2}t^{2}}$, the impact of this term becomes negligible, because under such circumstances the Wiener trajectory is almost monotone, and the CDF of the FHT can be approximated by

(12) $$F_{T}\left(t\right)\;\dot{=}\;1-\Phi \left(\frac{\omega -\mu t}{\sqrt{\sigma_{w}^{2}t+\sigma^{2}t^{2}}}\right)=\Phi \left(\frac{\mu t-\omega }{\sqrt{\sigma_{w}^{2}t+\sigma^{2}t^{2}}}\right)$$

Finally, differentiation of $F_{T}\left(t\right)$ with respect to *t* yields the corresponding PDF:

(13) $$f_{T}\left(t\right)\;\dot{=}\phi \left(\frac{\mu t-\omega }{\sqrt{\sigma_{w}^{2}t+\sigma^{2}t^{2}}}\right)\frac{\frac{\sigma_{w}^{2}}{2}\left(\mu t+\omega \right)+\omega \sigma^{2}t}{{\left(\sigma_{w}^{2}t+\sigma^{2}t^{2}\right)}^{3/2}}$$

where ϕ(·) denotes the standard normal PDF. According to the CDF of the FHT, the reliability function associated with Equation (12) can be directly derived as follows:

(14) $$R_{T}\left(t\right)=1-F_{T}\left(t\right)$$

Furthermore, the mean time to failure (MTTF) is given by

(15) $$MTTF=E\left[T\right]=\int_{0}^{\infty }R_{T}\left(t\right)dt$$

### 3.2. Quantification of Stress–Parameter Dependence

As discussed earlier, the stress–parameter mapping in ADT modeling is often determined empirically based on engineering judgment. Hence, developing a method to quantitatively characterize stress–parameter dependencies is essential for accurately evaluating product lifetime and reliability. To this end, we introduce the principle of acceleration factor invariance as a framework to quantify this relationship and present the following lemma.

**Definition 1**  ([33])**.** *Consider the lifetime distribution functions $F_{p}\left(t_{p}\right)$ and $F_{q}\left(t_{q}\right)$ under stress levels $S_{p}$ and $S_{q}$, respectively. If the following relation holds*

(16) $$F_{p}\left(t_{p}\right)=F_{q}\left(t_{q}\right),$$

*then the acceleration factor associated with the transition from $S_{p}$ to $S_{q}$ is given by*

(17) $$A_{p,q}=\frac{t_{q}}{t_{p}}.$$

In other words, the acceleration factor $A_{p,q}$ depends solely on the stress conditions, and does not vary with the specific observation time.

**Lemma 1.** 
*Based on Lemma 1, the invariance of the acceleration factor $A_{p,q}$ can be formally expressed by requiring equality of the lifetime CDFs obtained through the FHT representation. Employing Equation (12), the following characterization is derived:*

(18) $$\left\{\begin{array}{c} A_{p,q}={\left(\frac{\mu_{p}}{\mu_{q}}\right)}^{1/c}={\left(\frac{\sigma_{p}}{\sigma_{q}}\right)}^{1/c}={\left(\frac{\sigma_{wp}^{2}}{\sigma_{wq}^{2}}\right)}^{1/c}\\ c_{p}=c_{q} \end{array}\right.$$

A detailed derivation of Lemma 1 can be found in Appendix A. In the framework of ADT, the general form of the lifetime distribution for a product is assumed to remain unchanged, while its parameters are modulated by the applied stress conditions. This observation is consistent with Lemma 1. Equation (7) further demonstrates that the linear hypothesis is applicable to the three classical acceleration models. Following Lemma 1, it can be inferred that the parameters μ, σ and $\sigma_{w}$ in the ADT model (3) are stress-dependent, thereby supporting the validity of the linear hypothesis. Consequently, the following linear formulations are postulated for μ, σ and $\sigma_{w}$:

(19) $$\left\{\begin{array}{c} \ln\mu =a_{\mu }+b_{\mu }\varphi \left(S\right)\\ \ln\sigma =c_{\sigma }+d_{\sigma }\varphi \left(S\right)\\ \ln\sigma_{w}=u_{w}+v_{w}\varphi \left(S\right) \end{array}\right.$$

The coefficients $a_{\mu },b_{\mu },c_{\sigma },d_{\sigma },u_{w},v_{w}$ are unknown and must be estimated. In contrast, the parameters *c* are assumed to be unaffected by stress variation, and hence are excluded from the linear mappings. Collectively, the complete set of unknown quantities to be estimated is given by $\theta =\{a_{\mu },b_{\mu },c_{\sigma },d_{\sigma },u_{w},v_{w},c\}$. On the basis of Definition 1 and Lemma 1, the following theorem is established:

**Theorem 1.** 
*Consider the degradation process X(t) defined in Equation (1), which is modeled as an uncertainty-induced Wiener process characterized by the parameter set $\left\{a_{\mu },b_{\mu },c_{\sigma },d_{\sigma },u_{w},v_{w},c\right\}$. According to the structural relationships given in Equation (18), the following equalities hold among these parameters:*

(20) $$b_{\mu }=d_{\sigma }=2v_{w}.$$

A detailed justification of Theorem 1 is presented in Appendix B. Using Equation (20), one can further establish the explicit connections between the parameters $a_{\mu },b_{\mu },c_{\sigma },d_{\sigma },u_{w}$ and $v_{w}$, as shown below:

(21) $$\left\{\begin{array}{c} \ln\mu =a_{\mu }+b_{\mu }\varphi \left(S\right)\\ \ln\sigma =c_{\sigma }+b_{\mu }\varphi \left(S\right)\\ \ln\sigma_{w}=u_{w}+0.5b_{\mu }\varphi \left(S\right) \end{array}\right.$$

Accordingly, the set of unknown parameters requiring estimation, originally given by $\theta =\{a_{\mu },b_{\mu },c_{\sigma },d_{\sigma },u_{w},v_{w},c\}$, can be reduced to the reorganized form $\theta_{r}=\left\{a_{\mu },b_{\mu },c_{\sigma },u_{w},c\right\}$.

## 4. A Robust Parameter Estimation Method

In addition to the inherent stochasticity of degradation dynamics, external conditions such as environmental fluctuations and operational disturbances introduce further uncertainty into the modeling framework. These external influences not only affect parameter behavior under stress but also complicate the estimation procedure. Conventionally, parameter estimation is carried out after selecting the ADT model, from which lifetime distributions and reliability metrics under normal operating conditions can be inferred.

### 4.1. A Huber-Based Robust Parameter Estimation

In real-world scenarios, continuous monitoring in ADT is often impractical. Instead, periodic inspections are typically employed to assess the degradation status of the tested components. Under the constant-stress ADT (CSADT) framework, the degradation process can be described as follows: suppose there are *n* test units subjected to *L* distinct stress levels. Let $X(t_{kij})$ denote the *j*th observed degradation value for unit *i* under stress level *k*, recorded at inspection time $t_{kij}$, where k=1,2,…,L, $i=1,2,\ldots ,n_{k}$, and $j=1,2,\ldots ,m_{kj}$.

For the estimation procedure, maximum likelihood estimation (MLE), based on setting the derivatives of the log-likelihood to zero, is traditionally applied. Yet substituting Equation (21) into Equation (3) yields the likelihood function Equation (22), which has a highly entangled and nonlinear structure, rendering MLE computationally burdensome or even infeasible. More importantly, beyond computational complexity, MLE lacks robustness, preventing it from adequately addressing uncertainties arising from external factors. To overcome these challenges, we propose a two-step parameter estimation procedure, designed both to reduce the estimation burden and to improve robustness against external disturbances.

(22) $$L\left(x_{kij},t_{kij}\right)=\prod_{k=1}^{L}\prod_{i=1}^{n_{k}}\prod_{j=1}^{m_{ki}}\frac{1}{\sqrt{2\pi t_{kij}\left(\sigma^{2}t_{kij}+\sigma_{w}^{2}\right)}}\exp\left[-\frac{{\left(x-\mu t_{kij}\right)}^{2}}{2t_{kij}\left(\sigma^{2}t_{kij}+\sigma_{w}^{2}\right)}\right]$$

Within stochastic process frameworks, the expectation term reflects the systematic degradation pattern. For a Wiener process X(t)=X(0)+μt+σB(t), the drift component μt embodies the deterministic trajectory, while the diffusion term σB(t) accounts for stochastic perturbations. When the product exhibits stable deterioration, μ quantifies the average degradation velocity: μ>0 implies progressive wear, whereas μ<0 suggests a tendency towards recovery. Such trend information provides the foundation for evaluating reliability and forecasting remaining service life. Parameter estimation in Wiener trend begins with the identification of historical degradation trends. Since all units under the same stress share identical observation times $t_{kj}$, namely $t_{k1j}=t_{k2j}=\cdots =t_{kij}$, the trend at each inspection point can be represented by the mean value across the samples:

(23) $${\tilde{\mu }}_{kj}=\frac{1}{n_{k}}\sum_{i=1}^{n_{k}}X(t_{kij})$$

When Equation (21) is substituted into Equation (22), the parameters requiring estimation can be written as $\theta_{1}=\left\{a_{\mu },b_{\mu },c\right\}$. To address the robustness of the degradation process, a Huber-based robust loss function is carried out by replacing the quadratic loss function of the least squares, which minimizes the Huber-based function between empirical observations and the corresponding model-based predictions. The predicted mean takes the following form:

(24) $${\hat{\mu }}_{kj}=\exp\left(a_{\mu }+b_{\mu }\varphi \left(S\right)\right)\Lambda \left(t_{kij}\right)$$

In Equation (22), $\Lambda (t_{kij})$ is adopted as the representation of $t_{kij}$. Nevertheless, to simplify notation, the symbol $t_{kij}$ will still be used in the expression. Accordingly, the residual associated with the mean value can be written as follows:

(25) $$r_{kj}={\tilde{\mu }}_{kj}-{\hat{\mu }}_{kj}$$

Then, the Huber loss function can be expressed as follows:

(26) $$\rho_{\delta }\left(r\right)=\left\{\begin{array}{c} \frac{1}{2}r^{2},\;\;\;\;\left|r\right|\leq \delta ,\\ \delta |r|-\frac{1}{2}\delta^{2},\;\;\left|r\right|>\delta , \end{array}\right.$$

where δ>0 is the threshold. The objective function can be obtained:

(27) $$\arg\min L=\sum_{k=1}^{L}\sum_{j=1}^{m_{ki}}\rho_{\delta }\left(r_{kj}\right)$$

In order for the Huber loss to maintain reasonable efficiency under normal noise, it is necessary to robustly estimate the scale of the residuals. Let the set of all observed residuals be $\left\{r_{kj}\right\}$, and let the scale be estimated by the median absolute deviation (MAD):

(28) $$\hat{\delta }=1.4826\cdot {\mathrm{median}}_{k,j}\;\left(\;\left|r_{kj}-{\mathrm{median}}_{k,j}\left(r_{kj}\right)\right|\;\right).$$

where the constant $1.4826=1/\Phi^{-1}\left(0.75\right)$ serves as a correction factor, ensuring that the estimator is unbiased under a Gaussian distribution. The Huber threshold is then defined as $\delta =1.345\;\hat{\delta }$, where the value 1.345 is conventionally recommended to achieve an asymptotic efficiency of approximately 95% under normality. Then, one can optimize the parameters, ensuring convergence to the values that minimize *L*.

### 4.2. The Other Parameters Estimation Considering the Internal Uncertainty

In the first stage of estimation, three unknown parameters of the ADT model have already been identified. The remaining location parameters to be estimated are denoted by $\theta_{2}=\left\{c_{\sigma },u_{w}\right\}$, which appear in the maximum likelihood formulation of the model. The complete parameter vector is therefore given by $\theta_{r}=\theta_{1}\cup \theta_{2}$, where $\theta_{1}=\left\{a_{\mu },b_{\mu },c\right\}$ corresponds to the parameters obtained in the initial stage. Internal uncertainty is generally divided into two categories: that arising from heterogeneity and that arising from small sample sizes. In view of these uncertainties, it becomes necessary to further estimate the remaining parameters.

Although MLE is commonly applied to estimate all parameters simultaneously, its direct use in the proposed model is problematic. The large number of parameters increases the risk of estimation bias and can compromise the accuracy of lifetime and reliability predictions. The staged estimation strategy alleviates this issue by simplifying the parameter space and reducing the complexity of the subsequent estimation task.

While MLE remains effective for simpler stochastic process models, its application to the current ADT framework is hindered by the nonlinear structure of Equation (22), which includes terms such as $\sigma =\exp(c_{\sigma }+b_{\mu }\varphi \left(S\right))$. Evaluating the likelihood in this case would require working with cumbersome partial derivatives, leading to high computational cost, low efficiency, and in some instances, the absence of closed-form solutions. For models of this complexity, the Metropolis–Hastings (M-H) approach has become a practical and widely adopted alternative [5]. Let π(·) denote the posterior density of interest. By Bayes’ theorem, the joint posterior distribution can be expressed as

(29) $$\pi \left(\theta_{2}|x_{kij},t_{kij}\right)\propto L\left(\theta |x_{kij},t_{kij}\right)\;p\left(\theta_{2}\right),$$

where $L(\theta_{2}|x_{kij},t_{kij})$ is the likelihood function and $p(\theta_{2})$ represents the joint prior. The prior can be factorized into independent components:

(30) $$p\left(\theta_{2}\right)=p\left(c_{\sigma }|c_{1},c_{2}\right)\;p\left(u_{w}|u_{1},u_{2}\right).$$

Assuming Gaussian priors for all parameters (for example, $c_{\sigma }∼N\left(c_{1},c_{2}\right)$), the M-H sampler is employed. At each iteration, a candidate $\theta^{\prime }$ is drawn from a proposal distribution $K(\theta_{2}^{\prime }|\theta_{2})$. The move from the current state θ to the candidate $\theta^{\prime }$ is accepted with probability

(31) $$\vartheta =\min\left\{1,\frac{\pi \left(\theta_{2}^{\prime }\right)\;K\left(\theta_{2}|\theta_{2}^{\prime }\right)}{\pi \left(\theta_{2}\right)\;K\left(\theta_{2}^{\prime }|\theta_{2}\right)}\right\}.$$

This acceptance rule guarantees that the stationary distribution of the constructed Markov chain is the desired posterior $\pi (\theta_{2})$. The algorithm is initialized from the prior distribution and evolves by repeatedly applying the acceptance–rejection step. Over successive iterations, the chain traverses the parameter space and converges in distribution to $\pi (\theta_{2})$. Details of the implementation and theoretical properties can be found in Algorithm 1 and related references such as [18].
**Algorithm 1** M-H sampling for parameter inference.**Input:** Observed data $\left\{x_{kij},t_{kij}\right\}$; target posterior $\pi (\theta_{2})$; proposal kernel $K(\theta_{2}^{\prime }|\theta_{2})$.**Initialization:** Choose initial state $\theta_{2}\left(0\right)$.**for** ir=1,2,3,… **do**    Draw a candidate $\theta_{2}^{\prime }$ from $K(\cdot |\theta_{2}\left(ir\right))$.    Compute the acceptance probability ϑ using Equation (31).    Generate r∼Uniform(0,1).    **if** r<ϑ **then**          Accept: set $\theta_{2}\left(ir+1\right)=\theta_{2}^{\prime }$.    **else**          Reject: set $\theta_{2}\left(ir+1\right)=\theta_{2}\left(ir\right)$.    **end if****end for**

The reliability assessment of accelerated degradation tests is often challenged by the intrinsic statistical uncertainty that arises when only a limited number of samples are available. Such insufficiency in data size restricts the accuracy of model identification, thereby exerting a direct impact on subsequent evaluations of product reliability and lifetime prediction. To mitigate this limitation, interval estimation techniques are employed, which enable the construction of confidence intervals (CIs) for the fitted degradation model. This approach not only broadens the interpretative range of the estimated degradation trajectory but also enhances the robustness and credibility of the proposed hybrid modeling framework. In this study, we adopt a 95% confidence interval as the standard criterion. After obtaining point estimates of the model parameters, their corresponding mean values and variances are calculated. Based on these statistical quantities, the boundary values of the 95% confidence interval are determined using the conventional formulation for populations that follow a Gaussian distribution, under the significance level α=0.05.

(32) $$\left\{\begin{array}{c} \theta_{2}^{L}=\mathrm{Mean}-z\times \left(\frac{\mathrm{Standard}\mathrm{Deviation}}{\sqrt{n}}\right)\\ \theta_{2}^{U}=\mathrm{Mean}+z\times \left(\frac{\mathrm{Standard}\mathrm{Deviation}}{\sqrt{n}}\right) \end{array}\right.$$

where $\theta_{2}^{L}$ and $\theta_{2}^{U}$ denote the lower and upper bounds of the 95% confidence interval, respectively. The term *z* corresponds to the critical value of the standard normal distribution associated with the selected confidence level (for 95%, z≈1.96). The symbol *n* indicates the sample size, which in this study is fixed at n=186.

## 5. Case Study for the Electrical Contacts Degradation

**Figure 2:**
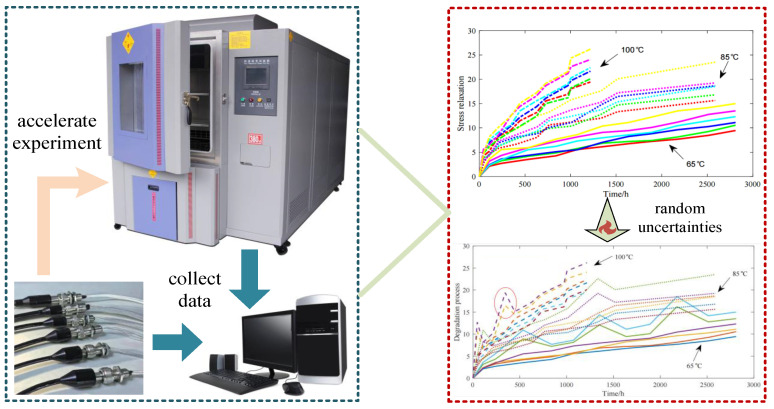
Electrical connectors’ degradation data under accelerated stresses.

Electrical connectors are essential elements in electric energy storage systems, providing the pathways that link storage units in both series and parallel arrangements. Their stability is crucial for ensuring reliable current transfer during charge and discharge operations. If a connector fails, the current path is interrupted, which may cause instability of the system, accelerated deterioration of the cells, and ultimately premature system failure. One of the key degradation mechanisms of connector contacts is stress relaxation, where materials gradually lose the ability to maintain stress under constant strain. To evaluate this mechanism, ADT under a constant-stress framework is conducted. In this setting, three elevated temperature levels are employed, namely 65 °C, 85 °C, and 100 °C, as shown in Figure 2. Six test units are subjected to these stresses, with inspections scheduled at fixed times $t_{kij}$, and at each inspection the stress relaxation measurement $x_{kij}$ is recorded, where $j=1,2,\ldots ,m_{ki}$ denotes the number of observations for unit *i* under stress level $S_{k}$. The reference stress level is 40 °C, while failure is defined once the stress relaxation exceeds 30% (*ω* = 30%). The dataset for this analysis originates from [34], and the summary results are listed in Table 1.

To further capture the variability observed in practical testing, random uncertainties caused by external factors are deliberately introduced into the original dataset. Specifically, they are inserted into three units tested at 65 °C (measurements three, six, and nine for the fifth and sixth units), two units at 85 °C (measurements two and eight for the fourth and fifth units), and two units at 100 °C (measurements one and four for the fourth and fifth units). The inclusion of such anomalies reflects random disturbances that could occur in practice and highlights the stochastic characteristics of degradation in electrical connector contacts, as shown in Figure 2.

### 5.1. Estimation of the Parameters

To identify the unknown parameters of the ADT model, a sequential estimation strategy is adopted. In the initial stage, the parameters associated with the Huber-type robust formulation are obtained, and in the subsequent stage, the remaining unknown quantities are determined. The numerical outcomes of this procedure are summarized in Table 2.

In particular, Table 2 reports the estimates for $\theta_{1}$. When carrying out the simulation, it is necessary to verify that the fitted values obtained from the observed data exhibit good agreement with the actual model parameters, thereby confirming the validity of the estimation process.

**Figure 3:**
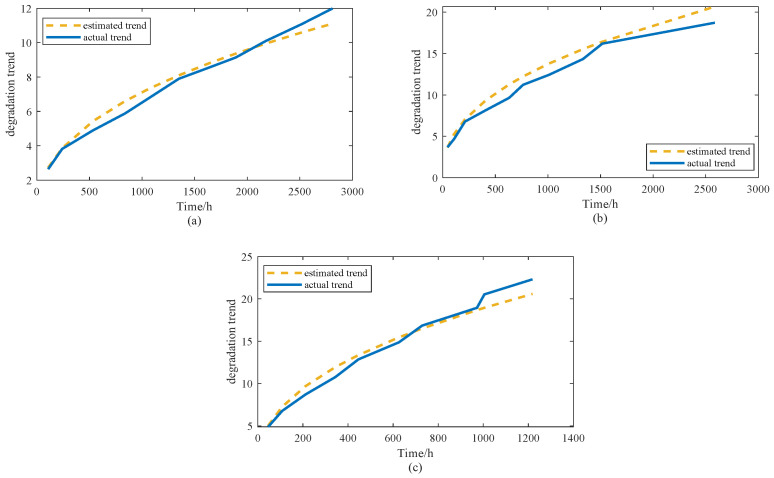
The estimated trend and the actual trend under (**a**) 65 °C, (**b**) 85 °C, (**c**) 100 °C stress levels.

To assess how well the estimated model-to-trend parameters capture the underlying degradation behavior, the obtained estimates are substituted into Equation (25) to generate the predicted trend curves for each stress condition. For clarity, Figure 3 illustrates a side-by-side comparison between the predicted trajectories and the actual observed degradation paths of the product.

Figure 3 presents a comparison between the estimated degradation trends and the actual observed degradation data under three accelerated stress conditions. At 65 °C, the estimated curve fits the empirical data well, showing that the model can effectively capture the relatively gradual and smooth degradation pattern occurring at lower stress levels. Only minor deviations are observed, suggesting that the estimation remains stable throughout the time horizon.

When the stress level is increased to 85 °C, the degradation process becomes noticeably faster. The estimated trend is able to follow this accelerated deterioration and provides a close approximation to the actual measurements. Although the observed data exhibit some fluctuations, the predicted trajectory still captures the overall progression of degradation with good accuracy.

At 100 °C, the degradation intensifies substantially, leading to a much sharper trend compared with the other two cases. Even under this extreme stress level, the estimated curve remains consistent with the empirical observations, indicating that the proposed method is capable of modeling both the rate and the magnitude of degradation. Overall, the figure demonstrates that the estimated model reproduces the observed degradation behavior reliably across different stress levels, thereby validating its applicability for accelerated degradation testing and lifetime prediction.

After the trend parameters have been obtained through the Huber-based approach, attention is directed toward estimating the other parameters in the ADT framework. These two groups of parameters are interdependent, since the trend estimates appear in Equation (22) during the evaluation of the likelihood function. The second phase of the estimation strategy is therefore carried out with the M-H algorithm. Table 3 presents the outcomes of this procedure, reporting quantities such as posterior mean, variance, Monte Carlo error, and the posterior quantiles (2.5%, median, 97.5%). Such statistical summaries provide a reliable basis for further investigations of lifetime prediction and reliability evaluation.

**Figure 4:**
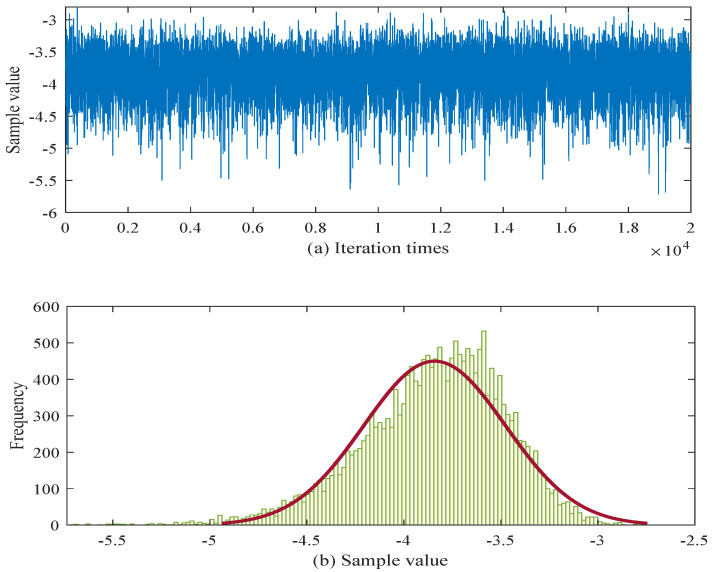
M-H sampling results for parameter $c_{\sigma }$.

**Figure 5:**
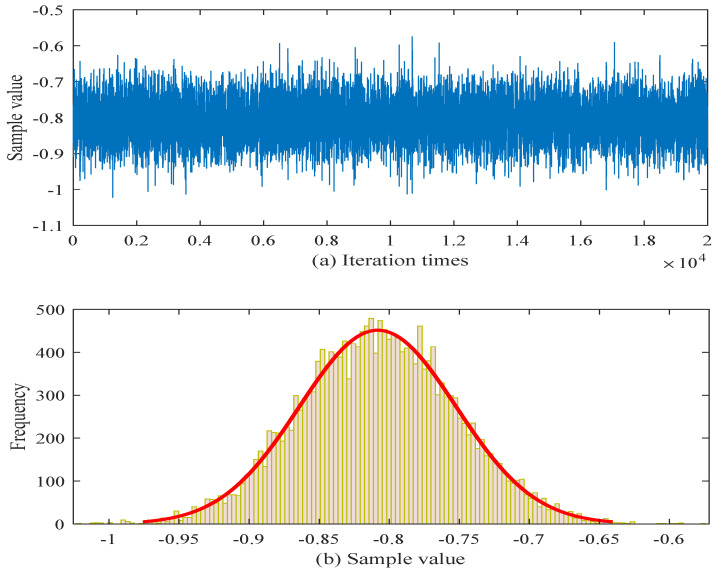
M-H sampling results for parameter $u_{w}$.

In order to provide a more intuitive view of the parameter estimation results, the posterior samples after a burn-in of 2000 iterations are illustrated in Figure 4 and Figure 5.

Figure 4 corresponds to the parameter $c_{\sigma }$. The trace plot shows that after burn-in, the chain fluctuates steadily within a bounded region (from approximately −5.5 to −3.0), without noticeable drift, which indicates satisfactory convergence and acceptable mixing of the Markov chain. The associated histogram is unimodal and relatively concentrated, with the bulk of the posterior mass centered around the mid-−4 range. This suggests that the M–H sampler has produced a stable estimate of $c_{\sigma }$ with moderate posterior uncertainty. Figure 5 depicts the results for $u_{w}$. The trace exhibits a similarly stable pattern, oscillating within a narrower interval (roughly between −1.1 and −0.6) and showing no signs of poor mixing or long-range dependence. The corresponding histogram is sharply peaked and more concentrated than that of $c_{\sigma }$, with most of the posterior mass around −0.8 to −0.7. This indicates that the posterior distribution of $u_{w}$ is well identified and exhibits lower variance.

Both figures indicate that, after the 2000-iteration burn-in, the M–H algorithm produces stable chains and unimodal posterior distributions for $c_{\sigma }$ and $u_{w}$, providing evidence that the estimation procedure is reliable for these parameters. To mitigate the internal uncertainty associated with limited sample sizes, a parameter estimation approach based on interval estimation is adopted. Consequently, according to Equation (32), the estimated values obtained from the interval estimation framework are summarized in Table 4.

To enable comparison with the proposed model, we also include a traditional Wiener process-based model that ignores the effect of internal uncertainties; in this case, σ is set to zero, and the model is denoted as M1. In addition, we examine the other scenarios introduced in Section 2. For case (1), the subcases (a)–(c) are labeled M2, M3, and M4, respectively. For case (2), the subcases (a)–(c) are denoted M5, M6, and M7, respectively. For case (3), the model is referred to as M8. These baseline models are incorporated into the case study for benchmarking purposes. Then, the estimated parameters’ values of these models are listed in Table 5.

According to Table 2 and Table 5, although the sets of parameters to be estimated differ, the results for the time-transformation parameter *c* are quite similar across all models. Hence, *c* can be considered independent of stress, which aligns with our earlier assumption. Table 2, Table 3, Table 4 and Table 5 present the parameter estimates obtained from different modeling approaches applied to the same degradation dataset. Variations in the estimated values arise from differences in model formulation and the treatment of uncertainty, which in turn affect the scale and shape of the fitted distributions. Nevertheless, parameter estimates alone cannot fully determine which model provides the most accurate representation of connector degradation under accelerated testing with uncertainties. A broader evaluation of model performance is therefore necessary to assess their comparative suitability.

**Figure 6:**
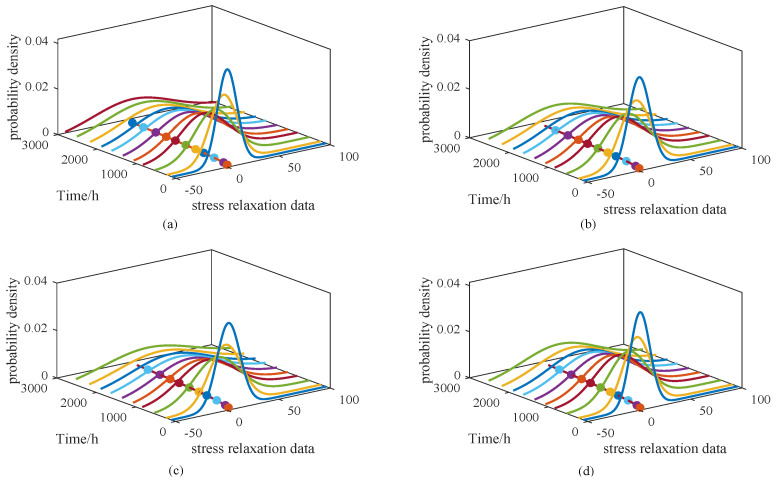
PDF curves and degradation trends of ADT data under 65 °C stress level for models (**a**) M1, (**b**) M2, (**c**) M3 and (**d**) M4.

**Figure 7:**
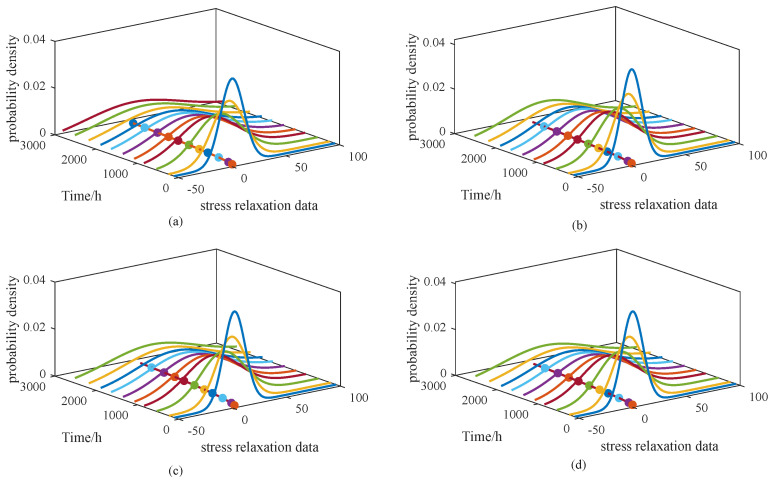
PDF curves and degradation trends of ADT data under 65 °C stress level for models (**a**) M5, (**b**) M6, (**c**) M7 and (**d**) M8.

Figure 6 and Figure 7 illustrate the probability density functions (PDFs) for models M1–M4 and M5–M8, respectively. To simplify the presentation, results are shown only for the 65 °C condition. Alongside the PDFs, the corresponding degradation trajectories are also plotted, enabling direct visual comparison of the models under consistent parameter settings.

**Figure 8:**
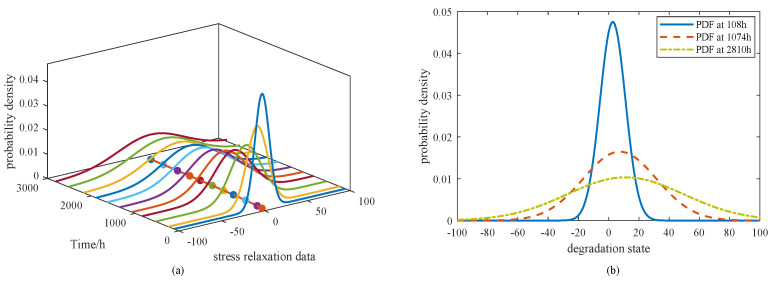
PDF curves of proposed model under 65 °C stress level for (**a**) PDF curves and degradation trend and (**b**) PDF curves at various measurement times.

From Figure 6, it can be observed that the PDFs of M1 and M4 are relatively narrow and peaked, whereas those of M2 and M3 appear broader, suggesting that the degradation behavior in M1 and M4 is more concentrated. In Figure 7, the PDF of M5 is noticeably wider than those of the other three models. Such broader distributions imply a more dispersed degradation process, corresponding to a larger variance. However, the overall degradation trends remain consistent, showing no substantial differences. To further illustrate the performance of the proposed approach, Figure 8 presents the PDF of the model under a stress level of 65 °C, along with the PDF curves at three sampling times: 108 h, 534 h, and 1074 h.

From Figure 8a, the probability density functions (PDFs) of the proposed model are shown to closely follow the overall degradation trajectory under the 65 °C stress condition. The degradation trend is effectively captured, and the fitted PDFs provide a clear representation of the probabilistic distribution of degradation states across the time horizon. This demonstrates that the model not only traces the mean degradation path but also quantifies the associated uncertainty through the shape and spread of the PDFs.

Figure 8b presents the PDFs at selected sampling times (108 h, 1074 h, and 2810 h). At the initial stage (108 h), the distribution is narrow and sharply peaked, reflecting a concentrated degradation state with low variance. As time advances, the distributions become wider, showing greater dispersion and increasing uncertainty. These observations confirm that the proposed model can dynamically capture both the central tendency and the variability of degradation behavior over time, which is essential for reliable lifetime prediction and accurate reliability assessment.

To provide stronger evidence of the distinctions observed in the figure, a quantitative evaluation of model performance was carried out. The computed metrics are reported in the next section to compare model accuracy across operational conditions.

### 5.2. Lifetime and Reliability Analysis

The primary goal of ADT is to evaluate product lifetime and reliability under nominal operating conditions. Achieving this requires converting stress-dependent parameters into their counterparts at the normal stress level. Accordingly, Equation (21) is recalculated under the condition of normal stress, with the transformation expressed as follows:

(33) $$\left\{\begin{array}{c} \ln\mu =a_{\mu }+b_{\mu }\varphi \left(S_{0}\right)\\ \ln\sigma =c_{\sigma }+b_{\mu }\varphi \left(S_{0}\right)\\ \ln\sigma_{w}=u_{w}+0.5b_{\mu }\varphi \left(S_{0}\right) \end{array}\right.$$

where $S_{0}$ denotes the nominal operating stress level given in Table 1. Because the parameter *c* does not vary with stress, it remains unchanged and requires no transformation.

**Figure 9:**
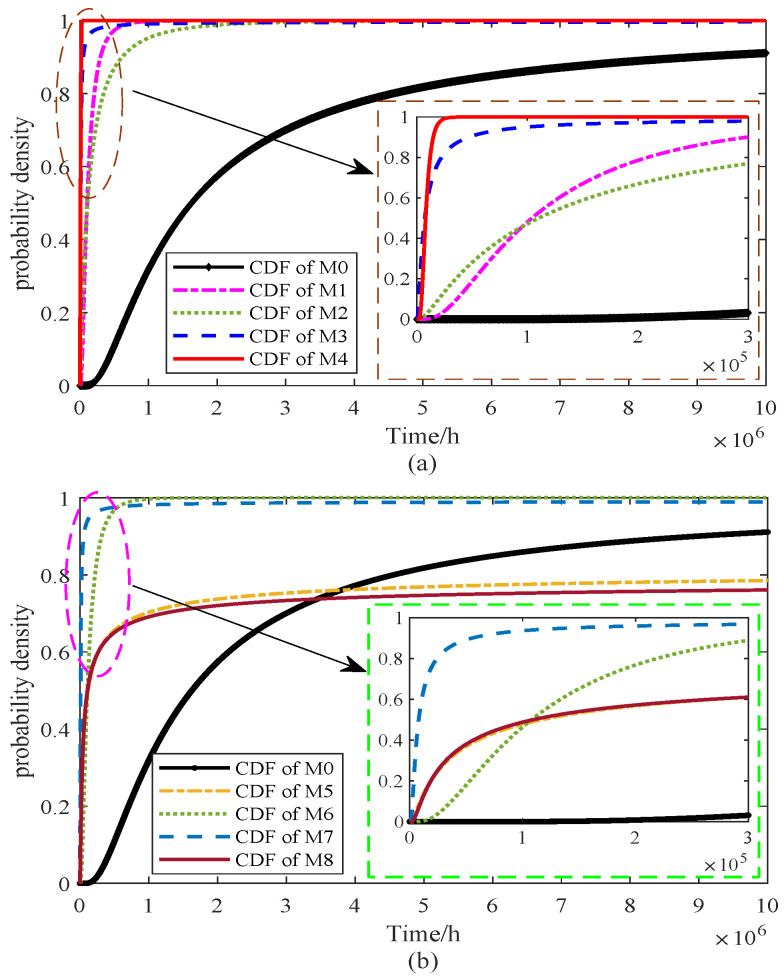
CDF of FHT for the models of (**a**) M0–M4 and (**b**) M0, M5–M8.

From Equations (33) and (12), the cumulative distribution function (CDF) of the FHT under nominal stress conditions can be directly derived. By substituting Equation (33), the closed-form expressions are obtained, and the corresponding CDF curves are plotted in Figure 9. For clarity of discussion, the models are organized into two groups, each including the proposed approach for comparison. In this context, the proposed model is denoted as M0.

**Figure 10:**
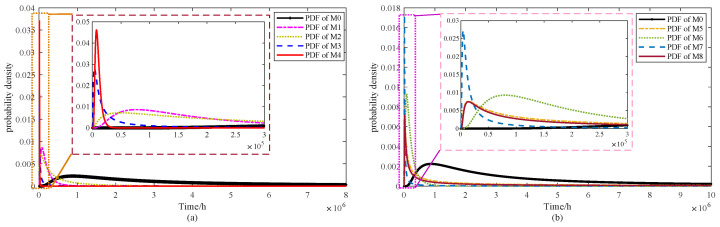
PDF of FHT for the models of (**a**) M0–M4 and (**b**) M0, M5–M8.

From Figure 9a, it is evident that the cumulative distribution functions (CDFs) of models M1–M4 differ noticeably from that of the proposed model M0. Specifically, the CDFs of M3 and M4 rise more steeply, implying shorter predicted lifetimes and smaller variances, while M1 and M2 exhibit relatively flatter curves, indicating longer tails and greater dispersion in the estimated lifetimes. In Figure 9b, the comparison of M0 with models M5–M8 underscores the differing stress–parameter mapping assumptions. The CDF curve of M5 is shifted left relative to M8, indicating earlier failure predictions, while M6 and M7 produce narrower distributions, suggesting reduced variability and a tendency to underestimate uncertainty. By contrast, M8 shows the slowest rise, corresponding to the longest predicted lifetime but potentially leading to an overestimation of reliability. Overall, the CDF of M0 does not rise the fastest nor the slowest; instead, it provides a balanced representation across the entire time horizon, supporting its suitability for characterizing lifetime distributions under nominal stress conditions. To further highlight the differences among the models, the PDFs derived from Equation (13) are presented in Figure 10.

**Figure 11:**
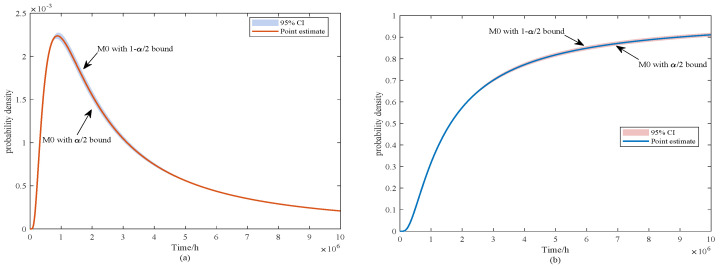
PDF and CDF of FHT for the M0 model under interval estimation (**a**) PDF and (**b**) CDF.

From Figure 10a, M1 and M3 exhibit sharp early peaks, suggesting shorter lifetimes with limited variability. In contrast, M2 and M4 produce flatter peaks, reflecting greater dispersion and delayed failures. In Figure 10b, the PDFs of M5–M8 are compared with M0. Both M5 and M8 display moderately wide peaks, suggesting moderate but not excessive variability. Model M6 yields the broadest distribution among them, whereas M7 shows a narrow and pronounced peak, indicating concentrated predictions with reduced uncertainty. M0 is distinctive in presenting the widest distribution overall, capturing the largest uncertainty range while remaining consistent with the degradation trend. This underscores the strength of the proposed model in representing both variability and realism in lifetime prediction. In addition, the PDF and CDF of M0 obtained through confidence interval estimation are illustrated in Figure 11.

From Figure 11, the point-estimated probability density function (PDF) of model $M_{0}$ exhibits a distinct unimodal shape, with the 95% confidence band being relatively narrow around the peak and gradually widening toward the tails. This indicates that the central part of the lifetime distribution is estimated with high stability, whereas predictions at the extremes are more sensitive to uncertainty. The corresponding cumulative distribution function (CDF) shows a similar pattern: the confidence interval remains tight around the median region (e.g., $t_{50}$), suggesting reliable estimation of central quantiles, while the interval widens toward the lower and upper tails, reflecting increased variability in early-failure and high-reliability quantiles (e.g., $t_{10}$ and $t_{90}$).

**Figure 12:**
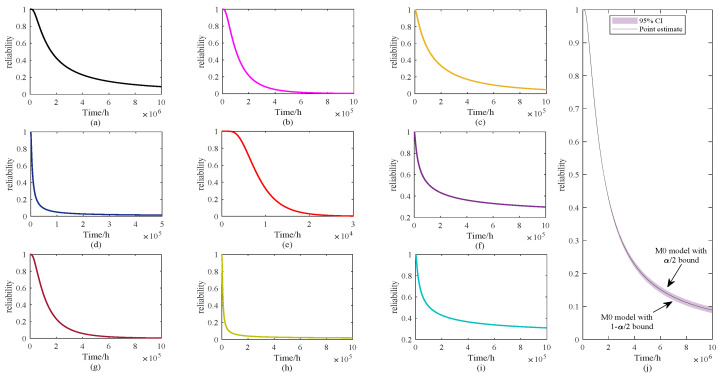
Reliability curves for the nine models (**a**) M0, (**b**) M1, (**c**) M2, (**d**) M3, (**e**) M4, (**f**) M5, (**g**) M6, (**h**) M7, (**i**) M8 and (**j**) interval estimation of M0.

It should be emphasized that the widening of the confidence intervals in the tail regions does not imply poor robustness of the proposed method. Robust estimation in this study is primarily applied to the mean degradation parameters, ensuring that the central trend remains stable even under data contamination. The interval estimation, on the other hand, addresses the additional uncertainty arising from limited sample sizes by explicitly quantifying its impact on variance-related parameters. Consequently, the combination of robust mean estimation and interval-based variance quantification allows $M_{0}$ not only to provide stable central predictions but also to transparently capture the sensitivity of extreme predictions, thereby enhancing the overall credibility of the lifetime analysis. Based on (14), the reliability curves of these models are shown in Figure 12.

Figure 12 presents the reliability curves of the nine models (M0–M8), where panel (a) and (j) correspond to the proposed method (M0) and its interval performance, respectively, and panels (b)–(i) display the baseline models. For a quantitative comparison, the median lifetime $T_{50}$ (defined as the time at which the reliability function R(t)=0.5) was approximated from the plots. The proposed model M0 and its interval values achieve the longest predicted lifetime with $T_{50}\approx \left(2.0\;-\;2.2\right)\times 10^{6}$ h. Among the baseline models, M8 also demonstrate relatively long lifetimes ($T_{50}\approx \left(1.5\;-\;1.8\right)\times 10^{5}$ h), though still significantly shorter than M0. In contrast, M3, M4 and M7 show the most rapid reliability decay, with $T_{50}$ values on the order of $10^{4}$ h, indicating much earlier predicted failures. The other models (M1, M2, M5, and M6) fall between these extremes, typically with $T_{50}$ in the range of $10^{4}$–$10^{5}$ h. The slope and spread of the curves further reflect model-specific uncertainty. The proposed method (M0) exhibits the broadest distribution with a gradual decline, capturing greater variability in the degradation process. Conversely, M3, M4 and M7 display steep drops, suggesting concentrated lifetime predictions that may underestimate uncertainty. M8 exhibits intermediate behavior, with reliability maintained longer than most baseline models, which may indicate an overestimation of reliability.

Overall, the results demonstrate that the proposed model M0 outperforms the baseline approaches by providing efficient lifetime estimates and effectively characterizing the uncertainty in the reliability distribution, thereby offering a more realistic representation of long-term degradation behavior.

To assess the quality of the model more rigorously, we conduct a quantitative evaluation. In the next section, these models are examined from multiple perspectives.

### 5.3. Model Comparison Results

**Figure 13:**
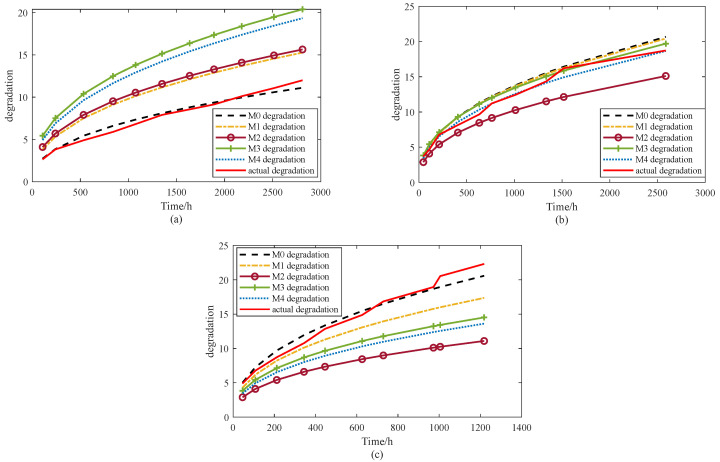
M0–M4 models’ degradation trends and actual degradation trend under stress levels of (**a**) 65 °C, (**b**) 85 °C, (**c**) 100 °C.

**Figure 14:**
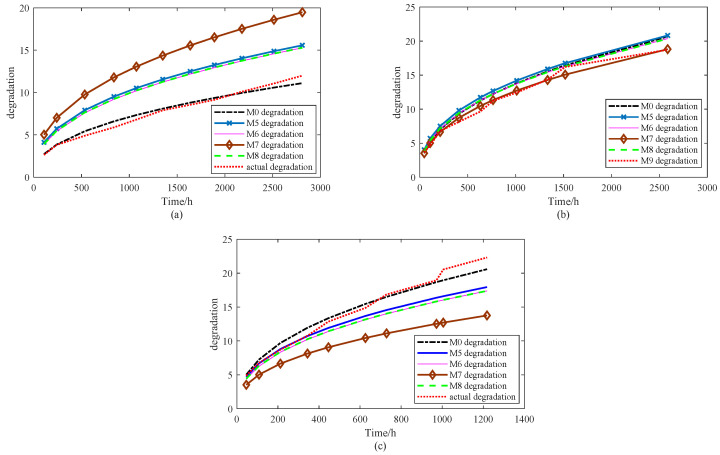
M0 and M5–M8 models’ degradation trends and actual degradation trend under stress levels of (**a**) 65 °C, (**b**) 85 °C, (**c**) 100 °C.

This section evaluates the nine models with respect to their capacity to capture degradation dynamics and predict product lifetime. As noted in Section 4, the mean function of a stochastic process provides a reliable description of the general degradation pattern. Therefore, the degree of agreement between the model-predicted mean trajectory and the experimental degradation data can be used as an indicator of model accuracy. Based on the parameter estimates listed in Table 2 and Table 5, Figure 13 and Figure 14 present a comparison of the mean degradation paths for M0 and the baseline models, offering a clearer assessment of how well each approach represents the underlying degradation behavior. For clarity, two figures are provided to compare the average degradation trends of models M0–M8 with the degradation data. Figure 13 presents the results for M0–M4, while Figure 14 illustrates the results for M5–M8.

As shown in Figure 13, under stress levels of 65 °C and 100 °C, model M0 provides the closest fit to the observed degradation trend. At 85 °C, the best agreement is observed for models M3 and M4, although M0 and M1 also demonstrate acceptable performance. At 65 °C, M3 exhibits the poorest fit, whereas M2 shows the weakest performance under both 85 °C and 100 °C. These results suggest that M2 fails to adequately represent the accelerated degradation process under uncertainty, which may in turn compromise the accuracy of lifetime predictions derived from this model.

Figure 14 illustrates that, consistent with the findings in Figure 13, the proposed model M0 delivers the best performance under the 65 °C and 100 °C stress conditions. At 100 °C, models M5, M6, and M8 approximate the observed degradation trajectory reasonably well during the initial 300 h; however, their accuracy declines as time progresses, suggesting that these models fail to adequately capture the underlying uncertainty. Under 85 °C, model M7 exhibits the closest fit to the degradation data, while the remaining models show comparable but slightly weaker performance. Nevertheless, M7 performs poorly at both 65 °C and 100 °C, lagging behind the others and highlighting its limited robustness.

In summary, visual inspection confirms that the proposed model M0 achieves the most consistent overall performance. It not only accounts for internal uncertainty within the modeling framework but also incorporates robust estimation techniques to mitigate the effects of external disturbances. As a result, M0 demonstrates superior adaptability and predictive accuracy compared with the baseline models. To further validate these conclusions, a quantitative evaluation is conducted using several widely adopted error metrics, including the root mean square error (RMSE), mean absolute error (MAE), relative average deviation (RAD), and relative standard deviation (RSD). These indices provide a rigorous basis for performance comparison across different stress levels, and the corresponding results are summarized in Table 6.

Table 6 presents the quantitative results of all models in terms of RMSE, MAE, RAD, and RSD. Overall, the proposed model M0 achieves the best accuracy, with the lowest RMSE (0.8751), MAE (0.6883), and RAD (0.066). These values are substantially smaller than those of the baseline models, confirming that M0 provides the most reliable approximation of the observed degradation behavior. Interestingly, M0 also yields the largest RSD (5.1109), which indicates that it captures a wider spread of variability while maintaining the lowest average errors. This balance suggests that M0 is not only accurate but also capable of representing the inherent uncertainty more comprehensively.

By contrast, models M2, M3, and M4 exhibit the poorest performance, with RMSE values exceeding 4.6 and MAE values above 3.6, as well as large RAD values (0.37–0.44). Although their RSD values are moderately high (3.95–4.96), the high error metrics imply that these models fail to effectively represent the accelerated degradation process. Models M1, M5, M6, and M8 perform better, with intermediate RMSE and MAE values in the range of 2.0–2.5 and RAD values around 0.22–0.24, but they still fall short of M0 in both accuracy and robustness. Model M7, despite showing comparable accuracy to M2–M4, suffers from instability across stress levels, as indicated by its high errors and relatively large RSD.

In summary, the results demonstrate that M0 consistently outperforms the competing models. While several baselines achieve moderate accuracy (M1, M5–M8), they either underestimate uncertainty or fail to capture the degradation dynamics under varying stress conditions. M2–M4, on the other hand, are clearly less reliable due to their high errors. Thus, M0 achieves the most favorable trade-off between accuracy and uncertainty representation, confirming its superiority in accelerated degradation modeling.

To complement the previous evaluation, two commonly used information criteria are applied: the Akaike Information Criterion (AIC) and the Bayesian Information Criterion (BIC) [35]. The AIC focuses on predictive capability by trading off model fit against the number of parameters, whereas the BIC imposes a stricter penalty on model complexity, especially as the sample size increases, and therefore often selects more parsimonious models to mitigate overfitting. By considering both measures, a balanced assessment of model adequacy can be achieved over the entire stress testing period. The resulting values, along with the mean time to failure (MTTF) estimates, are summarized in Table 7.

Table 7 reports the AIC, BIC, and MTTF values for all models. Among them, the proposed model M0 achieves the lowest AIC with interval (810.64 [810.3301, 811.0032]) and BIC with interval (817.09 [816.7816, 817.4547]), demonstrating the best overall trade-off between model fit and complexity. In contrast, several alternative models (e.g., M3, M4, and M7) yield considerably higher AIC and BIC values, suggesting inferior performance either due to poorer fit or over-parameterization. Regarding the mean time to failure (MTTF), M0 provides an estimate of $3.47\times 10^{6}$, which lies within a reasonable range under the accelerated testing framework. Models M5 and M8 report even higher MTTF values ($4.58\times 10^{6}$ and $5.03\times 10^{6}$, respectively). Although these results suggest longer predicted lifetimes, their larger AIC and BIC scores imply weaker model adequacy. From a reliability perspective, such elevated MTTF estimates may reflect an overestimation of product durability, leading to overly optimistic reliability predictions. Consequently, despite the longer MTTF reported by M5 and M8, M0 remains the most reliable and parsimonious model, offering both accurate lifetime characterization and robust model selection criteria performance.

## 6. Conclusions

This study proposes a robust accelerated degradation modeling framework built upon a Wiener-process formulation to confront multiple sources of uncertainty in reliability analysis. Internal variability is characterized by incorporating random effects together with interval estimation, which collectively capture the influence of heterogeneity and small sample sizes. External perturbations are mitigated through a hybrid inference procedure that integrates Huber-type loss with a Metropolis–Hastings algorithm, thereby reducing the impact of noise, outliers, and other disturbances. In addition, the accelerated factor constant principle is utilized to construct a quantitative stress–parameter linkage, enabling consistent extrapolation of degradation behavior from accelerated tests to nominal stress conditions. Application to stress relaxation data of electrical connectors verifies that the proposed methodology yields more stable parameter estimates and improves the accuracy of lifetime prediction under uncertain environments.

Future investigations may extend the present work by generalizing the stochastic formulation to encompass more complex degradation mechanisms, integrating online monitoring information for adaptive updating of reliability assessments, and refining stress-parameter mappings in multi-stress or time-dependent scenarios. In addition, a key objective is to develop comprehensive accelerated degradation models that analytically quantify uncertainty, account for non-stationary behaviors, and leverage stochastic processes such as Gamma and inverse Gaussian processes to enhance real-world applicability. These directions will broaden the applicability of stochastic accelerated degradation testing and further enhance its role in the reliability assurance of safety-critical systems.

## Figures and Tables

**Table 1:** Basic information under CSADTs of the stress relaxation.

Test Information	CSADT
Acceleration model	Arrhenius model
Stress levels	*S*_0_ = 40 °C, *S*_1_ = 65 °C,
	*S*_2_ = 85 °C, *S*_3_ = 100 °C,
	*S_H_* = 100 °C,
Number of stress levels	L=3
Sample size	$n_{k}=6$
	(under each stress level)
Number of measurements	$m_{1j}=11,m_{2j}=10$,
	$m_{3j}=10$
Failure threshold	ω=30

**Table 2:** Huber-based parameter estimation.

	$a_{\mu }$	$b_{\mu }$	*c*
$\theta_{1}$	−2.7556	2.7215	0.4305

**Table 3:** Estimation of remaining unknown parameters.

Model Parameters	Estimation Results					
	Mean	Variance	MC Errorh	2.5%	Median	97.5%
$c_{\sigma }$	−3.8415	0.1335	0.0046	−4.6429	−3.8072	−3.2255
$u_{w}$	−0.808	0.0031	0.1154	−0.9166	−0.8075	−0.6989

**Table 4:** Lower and upper bound estimates of the 95% confidence interval.

Parameters	$\theta_{2}^{L}$	$\theta_{2}^{U}$
$c_{\sigma }$	−3.8940	−3.7890
$u_{w}$	−0.816	−0.8

**Table 5:** The estimated values of M1-M8 models’ parameters.

	M1	M2	M3	M4	M5	M6	M7	M8
μ	#	#	0.8116	0.6913	#	#	0.7178	#
$a_{\mu }$	−1.5357	−1.3701	*	*	−1.3525	−1.4622	*	−1.438
$b_{\mu }$	1.3544	1.3361	*	*	1.3443	1.3358	*	1.3295
σ	0	0.0012	#	0.0023	#	0.0004	#	#
$c_{\sigma }$	*	*	−1.266	*	−1.3186	*	−1.2267	−1.2652
$d_{\sigma }$	*	*	−0.9108	*	−0.9378	*	−0.9924	−1.0096
$\sigma_{w}$	#	−1.5507	1.6216	#	1.5672	#	#	#
$u_{w}$	−0.1062	*	*	−0.0774	*	−0.0696	−0.0648	−0.0748
$v_{w}$	0.5606	*	*	0.5885	*	0.5468	0.5869	0.5604
*c*	0.4273	0.4109	0.4059	0.4194	0.4075	0.4197	0.4156	0.4172

* indicates that no values are available. # denotes a linear stress–parameter function expression.

**Table 6:** Comparison results based on RMSE, MAE, RAD and RSD.

	RMSE	MAE	RAD	RSD
M0	0.8751	0.6883	0.066	5.1109
M1	2.4489	2.0151	0.2178	4.4101
M2	4.6399	3.8403	0.3684	3.9597
M3	4.9038	3.8810	0.4422	4.9615
M4	4.6882	3.6703	0.4019	4.6155
M5	2.5434	2.1205	0.2415	4.5289
M6	2.4697	2.0345	0.2226	4.3863
M7	4.7097	3.6824	0.4064	4.6522
M8	2.4774	2.0418	0.2244	4.3788

**Table 7:** Values of AIC, BIC and MTTF.

Models	AIC	BIC	MTTF ($\times 10^{6}$)
M0	810.6375	817.089	3.4706
M1	812.0407	828.1695	0.1452
M2	815.8865	832.0152	0.2572
M3	827.3062	843.4349	0.1129
M4	823.1377	839.2664	0.009
M5	818.6674	838.0218	4.5769
M6	814.3418	833.6962	0.152
M7	825.917	845.2715	0.2536
M8	817.2446	839.8248	5.0302

## Data Availability

The data presented in this study will be made available on request to the corresponding author after obtaining permission from an authorized person.

## Appendix A. Proof of Lemma 1

According to Definition 1, inserting Equation (17) into Equation (16) gives $F_{p}\left(t_{p}\right)=F_{q}\left(A_{p,q}t_{p}\right)$. Next, by substituting Equation (12) into this relation and setting Λ(t)=t, we arrive at

(A1) $$\Phi \;\left(\frac{\mu_{p}t_{p}-\omega }{\sqrt{\sigma_{p}^{2}t_{p}^{2}+\sigma_{wp}^{2}t_{p}}}\right)=\Phi \;\left(\frac{A_{p,q}\mu_{q}t_{p}-\omega }{\sqrt{A_{p,q}^{2}\sigma_{q}^{2}t_{p}^{2}+A_{p,q}\sigma_{wq}^{2}t_{p}}}\right).$$

For Equation (A1) to hold for arbitrary $t_{p}$, the corresponding conditions must satisfy

(A2) $$\left\{\begin{array}{c} \mu_{p}t_{p}-\omega =A_{p,q}\mu_{q}t_{p}-\omega ,\\ \sigma_{p}^{2}t_{p}^{2}+\sigma_{wp}^{2}t_{p}=A_{p,q}^{2}\sigma_{q}^{2}t_{p}^{2}+A_{p,q}\sigma_{wq}^{2}t_{p}. \end{array}\right.$$

From Equation (A2), one can directly deduce

(A3) $$A_{p,q}=\frac{\mu_{p}}{\mu_{q}}=\frac{\sigma_{p}}{\sigma_{q}}=\frac{\sigma_{wp}^{2}}{\sigma_{wq}^{2}}.$$

It follows from Equation (A2) that since $A_{p,q}\neq 1$ for p≠q, the parameters μ, σ, and $\sigma_{w}$ must vary with stress. If the time-scaling function is generalized to $\Lambda \left(t\right)=t^{c}$, the analogous derivation yields

(A4) $$\left\{\begin{array}{c} A_{p,q}={\left(\frac{\mu_{p}}{\mu_{q}}\right)}^{1/c}={\left(\frac{\sigma_{p}}{\sigma_{q}}\right)}^{1/c}={\left(\frac{\sigma_{wp}^{2}}{\sigma_{wq}^{2}}\right)}^{1/c},\\ c_{p}=c_{q}=c. \end{array}\right.$$

This concludes the proof.

## Appendix B. Proof of Theorem 1

The dependence of the model parameters on the accelerated stress, given in Equation (7), implies that for the $p^{th}$ and $q^{th}$ stress levels, $\varphi (S_{p})$ and $\varphi (S_{q})$, the parameters μ, σ, and $\sigma_{w}$ can be expressed as

(A5) $$\left\{\begin{array}{c} \ln\mu_{p}=a_{\mu }+b_{\mu }\varphi \left(S_{p}\right),\\ \ln\mu_{q}=a_{\mu }+b_{\mu }\varphi \left(S_{q}\right), \end{array}\right.$$

(A6) $$\left\{\begin{array}{c} \ln\sigma_{p}=c_{\sigma }+d_{\sigma }\varphi \left(S_{p}\right),\\ \ln\sigma_{q}=c_{\sigma }+d_{\sigma }\varphi \left(S_{q}\right), \end{array}\right.$$

(A7) $$\left\{\begin{array}{c} \ln\sigma_{wp}=u_{w}+v_{w}\varphi \left(S_{p}\right),\\ \ln\sigma_{wq}=u_{w}+v_{w}\varphi \left(S_{q}\right). \end{array}\right.$$

By subtracting the *p*th expression from the *q*th in Equations (A5)–(A7), and recalling the definition of $A_{p,q}$, we obtain

(A8) $$b_{\mu }\left(\varphi \left(S_{p}\right)-\varphi \left(S_{q}\right)\right)=d_{\sigma }\left(\varphi \left(S_{p}\right)-\varphi \left(S_{q}\right)\right)=2v_{w}\left(\varphi \left(S_{p}\right)-\varphi \left(S_{q}\right)\right).$$

Hence, Equation (A8) reduces to

(A9) $$b_{\mu }=d_{\sigma }=2v_{w}.$$

This concludes the proof.
